# Intravenous interleukin-2 in patients over 65 with metastatic renal carcinoma.

**DOI:** 10.1038/bjc.1992.152

**Published:** 1992-05

**Authors:** S. Négrier, A. Mercatello, M. Bret, P. Thiesse, J. Y. Blay, B. Coronel, Y. Merrouche, R. Oskam, C. R. Franks, M. Clavel

**Affiliations:** Department of Medical Oncology, Centre L. Bérard, Lyon, France.

## Abstract

The present study was designed in order to evaluate the response rate and the toxicity of continuous infusion of Interleukin 2 (IL2) in patients over 65 with metastatic renal cell carcinoma. Twenty-five patients, median age 69 (range 65-77), without any prior systemic anticancer therapy received a continuous infusion of IL2 at a dose of 18 x 10(6) iu m-2 d-1 for 2 periods of 5 days separated by a 6 day break. Toxicity was not different compared with younger patients (e.g. fever, hypotension, rise in creatinine level), except for cardiac toxicity which was of great concern. Despite normal cardiac tests prior to inclusion into the study, abnormalities of the cardiac rhythm ranging from tachycardia to ventricular extrasystoles occurred in 44% of the patients and IL2 cardiac toxicity was responsible for one toxic death. Three objective responses, i.e. one partial and two complete persistent responses, were seen in 22 evaluable patients. Thus, if age does not seem to modify the potential for response to IL2 therapy, cardiac toxicity appears as a limiting factor for intravenous schedules of IL2.


Br. J. Cancer (1992), 65, 723–726 © Macmillan Press Ltd., 1992

### Intravenous interleukin-2 in patients over 65 with metastatic renal carcinoma

S. Négrier[1], A. Mercatello[2], M. Bret[2], P. Thiesse[1], J.Y. Blay[1], B. Coronel[2], Y. Merrouche[1], R. Oskam[3], C.R. Franks[3], M. Clavel[1], J.F. Moskovtchenko[2] & T. Philip[1]

[1]*Department of Medical Oncology, Department of Radiology, Centre L. Bérard, 28 rue Laënnec, 69373 – Lyon Cedex 08;* [2]*Department of Intensive Care, Pavillon P, Hôpital E. Herriot, Place d'Arsonval, Lyon 69008, France;* [3]*Eurocetus B.V., Paasheuvelweg 30 1105 Amsterdam-Zuidoost, The Netherlands.*

**Summary** The present study was designed in order to evaluate the response rate and the toxicity of continuous infusion of Interleukin 2 (IL2) in patients over 65 with metastatic renal cell carcinoma. Twenty-five patients, median age 69 (range 65–77), without any prior systemic anticancer therapy received a continuous infusion of IL2 at a dose of $18 \times 10^6$ iu $m^{-2}$ $d^{-1}$ for 2 periods of 5 days separated by a 6 day break. Toxicity was not different compared with younger patients (e.g. fever, hypotension, rise in creatinine level), except for cardiac toxicity which was of great concern. Despite normal cardiac tests prior to inclusion into the study, abnormalities of the cardiac rhythm ranging from tachycardia to ventricular extrasystoles occurred in 44% of the patients and IL2 cardiac toxicity was responsible for one toxic death. Three objective responses, i.e. one partial and two complete persistent responses, were seen in 22 evaluable patients. Thus, if age does not seem to modify the potential for response to IL2 therapy, cardiac toxicity appears as a limiting factor for intravenous schedules of IL2.

Although renal cell carcinoma is a relatively rare tumour, its natural history has at least two remarkable points, i.e. the highest incidence occurs at 65 years of age and 50 to 60% of the patients develop distant metastases (Holland, 1977; Ritchie *et al.*, 1987). Until the eighties, attempts to treat widespread diseases were not successful and renal cancer is considered as a radio-resistant and chemoresistant tumour (Finney, 1973; Droz *et al.*, 1988).

Therefore, the 30% response rate, with 10% complete response in patients treated by intravenous recombinant Interleukin-2 (IL2) first reported by Rosenberg *et al.* (1987), was considered as a possible breakthrough in this disease. Further studies, though with a generally lower dose of IL2 and lower response rate, unequivocally confirmed the activity of IL2 in metastatic renal cell carcinoma (West *et al.*, 1987; Negrier *et al.*, 1990).

Nevertheless, transient toxic effects of IL2 therapy are unavoidable, and no predictive factor for response to IL2 therapy is known yet. Responding patients remain a minority. The justification of IL2 therapy in all patients is still a matter of debate. Using the West schedule (West *et al.*, 1987), we conducted a phase II trial of intravenous IL2 in metastatic renal cell carcinoma restricted to patients over 65 years of age. This study reports the characteristics of the patients, the toxic events and the responses observed with IL2 in this population.

### Patients and methods

### *Patients*

Between October 1987 and January 1991, 33 patients over 65 with metastatic renal cell carcinoma were referred to our institute. Eligible patients had to meet the following criteria: histologically documented evidence of metastatic renal cell carcinoma with measurable progressive disease, no prior chemotherapy or extensive radiotherapy in the last 4 weeks prior to registration, an ambulatory performance status (ECOG 0-1, Karnofsky ⩾80%), blood cell count, serum bilirubin and creatinine within the normal range, no evidence of brain metastases at CT scan. Patients were excluded if they had a significant history or current evidence of serious organ pathology.

Twenty five patients were eligible and eight were excluded. The main reasons of exclusion were: Karnofsky's score <80% (3), brain metastases (2), refusal (2), severe hypercalcemia (1). The age of excluded patients ranged from 66 to 75 (median 70). Excluded patients received palliative treatments and/or chemotherapy as phase II studies.

The characteristics of the 25 eligible patients are detailed in Table I. There were 16 males and nine females; median age was 69 (range 65–77). None of them had received prior treatment except surgery (23 nephrectomy/25).

All patients were required to give informed consent prior to participation in the study.

### *Supportive care and monitoring*

Before inclusion each patient underwent a cardiac evaluation which consisted in E.C.G. and ultrasound cardiography with determination of the left ventricular work index.

All patients were monitored daily during the therapy by physical examination and measurement of cardiac rhythm, blood pressure, temperature and weight. Full blood count and full biochemical analysis were also performed daily. A chest X-ray was required before treatment and was repeated twice weekly. All side effects were registered daily and evaluated according to the WHO criteria (Miller *et al.*, 1981). Additional treatment included systemic antibiotic (peflacin). Vascular filling was assumed by 20% albumin infusion in order to maintain central venous pressure within the normal range or when hypotension occurred. In case of persistent hypotension, albumin infusion was prolonged and dopamin was added in a progressive dosage (0.5 to 1.5 $\mu g\,k^{-1}\,min^{-1}$) until blood pressure correction. Fever was routinely treated by acetaminophen, and indomethacin was added in case of grade 4 fever.

### *Treatment*

Treatment protocol was reviewed and accepted by the ethical committee of University C. Bernard in Lyon.

Correspondence: T. Philip, Department of Medical Oncology, Centre L. Bérard, 69373 – Lyon Cedex 08, France.
This work was supported by the Association pour la Recherche sur le Cancer (ARC).
Received 14 August 1991; and in revised form 20 December 1991.

**Table I** Principal characteristics of the 25 patients at time of inclusion

| *Patient no.* | *Sex* | *Age* | *Prior nephrectomy* | *Number of tumour sites* | *Time between primary tumour and metastases (months)* |
|---|---|---|---|---|---|
| 1 | F | 70 | + | 3 | 6 |
| 2 | M | 69 | + | 2 | 72 |
| 3 | M | 77 | + | 2 | 0 |
| 4 | M | 72 | + | 3 | 0 |
| 5 | F | 73 | + | 2 | 9 |
| 6 | M | 67 | – | 2 | 0 |
| 7 | F | 66 | + | 1 | 0 |
| 8 | M | 65 | + | 3 | 0 |
| 9 | M | 66 | + | 1 | 60 |
| 10 | M | 73 | + | 2 | 18 |
| 11 | M | 66 | + | 1 | 0 |
| 12 | F | 69 | + | 2 | 0 |
| 13 | M | 69 | + | 2 | 0 |
| 14 | F | 67 | + | 2 | 6 |
| 15 | F | 69 | + | 3 | 0 |
| 16 | F | 67 | + | 3 | 0 |
| 17 | M | 72 | + | 3 | 0 |
| 18 | M | 70 | – | 3 | 0 |
| 19 | M | 68 | + | 1 | 3 |
| 20 | M | 77 | + | 2 | 72 |
| 21 | M | 71 | + | 1 | 6 |
| 22 | F | 69 | + | 2 | 0 |
| 23 | M | 68 | + | 1 | 84 |
| 24 | F | 69 | + | 1 | 24 |
| 25 | M | 72 | – | 2 | 0 |

Patients received rIL2 as a 5-day intravenous (i.v.) continuous infusion, at a dose of $18 \times 10^6$ iu m$^{-2}$ d$^{-1}$ followed by a 6-day break. A second 5-day continuous infusion at the same dose was started at the end of the rest period. After two identical induction cycles with 3 week rest in between, patients whose disease was at least stable received four maintenance cycles of rIL2 at $18 \times 10^6$ iu m$^{-2}$ d$^{-1}$ for 5 days. These cycles started 3 weeks following completion of the second induction cycle; each maintenance treatment was repeated every 4 weeks if progression did not occur.

Recombinant IL-2 was supplied by Eurocetus B.V. Amsterdam. The Netherlands. Its specific activity was $18 \times 10^6$ International Units per milligram of protein. The dry-frozen product was reconstituted with 1.2 ml of sterile water; 0.1 ml of the solution, corresponding to 1,800,000 iu IL-2, was administered via an electric pump through a standard subclavian catheter as a continuous infusion over 24 h, but the drug was prepared every 12 h by dilution in 50 ml of 5% dextrose. A close monitoring was performed during each cycle of therapy.

### *Evaulation of response to treatment*

Response was considered as complete when all measurable tumour had disappeared for a period of at least 1 month. Partial response was a decrease by at least 50% of the sum of the products of all measurable disease with no evidence of any new lesion. Progressive disease was defined as an increase of at least 25% in one of the measurable lesions or by any additional lesion. The absence of progression was verified between the first and second induction cycles by physical examination and chest X-ray. Evaluation was performed 3 weeks after the second induction cycle and 3 weeks after the last maintenance cycle.

### Results

### *Administration of IL2*

Among the 25 patients treated, 19 received a second induction course and six were given at least one maintenance cycle. The mean dose of $IL_2$ administered was of $15 \times 10^6$ iu m$^{-2}$ d$^{-1}$ (median dose: $18 \times 10^6$ iu m$^{-2}$ d$^{-1}$).

76% of patients received at least 70% of the planned dose during induction course 1 and 61% during course 2.

### *Toxicity*

All patients were evaluable for toxicity, and toxic events are summarised in Tables II, III and IV. One patient was withdrawn early from the study (after a 4-day infusion on first cycle) because of active infection and iatrogenic pneumothorax. One toxic death occurred in a patient of 65, at the end of the second period of the first induction course. This patient had normal cardiac tests prior to joining the study (E.C.G., ultrasonography). During IL2 therapy, important asthenia and hypotension (grade 3) were noted. Hypotension was controlled by dopamine infusion. On the last day of treatment repeated ventricular extrasystoles appeared but regressed after intravenous injection of amiodarone and IL2 interruption. Nevertheless, though metabolic and biochemical blood controls were normal, arrhythmia reoccurred, once as a supraventricular tachycardia (pulse 200 min$^{-1}$) and then as runs of extrasystoles. Both episodes were controlled again by amiodarone which was then administered as a continuous infusion. Three hours later, extrasystoles associated with hypotension, then a ventricular fibrillation occurred. Specific treatments (electric shock, epinephrine injection) failed to reduce this ventricular fibrillation and the patient died 12 h after cessation of IL2; the family refused the autopsy. Cardiac toxicity was noted in 11/25 (44%) of the patients and appeared mostly as cardiac rhythm abnormalities ranging from tachycardia ($n = 5$) to ventricular extrasystoles ($n = 6$). Three patients developed a grade 4 (WHO) hypotension which resolved after colloid and

**Table II** Hypotention and fever according to the WHO grades observed during all cycles of therapy

| | *WHO grades* | | | | | |
|---|---|---|---|---|---|---|
| | *1* | *2* | *3* | *4* | *Total* | *Percentage* |
| Hypotension | 1 | 6 | 11 | 3 | 23 | 92% |
| Fever | 1 | 13 | 6 | – | 25 | 100% |

For each patient, the maximum severity through the successive cycles is considered.

**Table III** Toxicities registered during all cycles of IL2 therapy other than fever and hypotension

| *Adverse event* | *Incidence (%)* |
|---|---|
| Erythema | 64 |
| Oliguria | 60 |
| Diarrhoea | 56 |
| Neurotoxicity | 52 |
| Nausea or vomiting | 52 |
| Pruritus | 48 |
| Cardiac rhythm disturbances | 44 |
| Dyspneoa | 28 |
| Oedema | 20 |
| Urinary retention | 8 |
| Cyanosis | 8 |
| Toxic death (1 patient) | 4 |

All these toxicities were evaluated as grade 1 to 3 (WHO).

**Table IV** Biological changes during all cycles of therapy according to the WHO grades

| | *WHO grades* | | | | | |
|---|---|---|---|---|---|---|
| *Parameter tested* | *1* | *2* | *3* | *4* | *Total* | *Percentage* |
| Creatinine | 5 | 9 | 3 | 1 | 18 | 72% |
| Bilirubin | 6 | 1 | 1 | – | 8 | 32% |
| SGOT | 7 | 1 | – | – | 8 | 32% |
| Hemoglobin | 5 | 6 | 5 | 1 | 16 | 64% |
| White blood cells | 2 | – | – | – | 2 | 8% |
| Platelets | 1 | 1 | – | – | 2 | 8% |

dopamine infusions in two cases and requested interruption of the therapy in one. Fever was present in all patients but routinely well controlled by acetaminophen; the use of indomethacin was restricted to a few cases (e.g. two patients). Gastro-intestinal signs of moderate severity were commonly observed; in three patients, however, vomiting and diarrhoea required dose modifications at almost each successive course. Weight gain below 5% was observed in 15 patients, but only two patients overloaded 10% of their basal weight.

Renal toxicity was of great concern with a rise of creatinine levels over $300\,\mu g\,l^{-1}$ in 18/25 patients but dose modification was required in a few cases only (i.e. 2/25). Hepatic and haematological disturbances were also frequent but always transient and moderate. Of note, the number of transfusional requirements was particuarlaly low, i.e. four red blood cell transfusions and no platelet transfusion.

Once treatment was completed all toxicities of rIL2 resolved within 1 to 2 weeks leaving no residual deficit.

*Response to therapy*

Three patients were not evaluable i.e. one early withdrawal (after 4 days of treatment for catheter-related complications), one toxic death and one patient who was considered a posteriori not to have significant measurable lesions (<1 cm). Evaluation of the 22 remaining patients showed one partial and two complete responses, giving an overall response rate of 3/25 with three responders in 22 evaluable patients. Details of responding patients are shown in Table V. In the two patients who are in complete remission a partial response (i.e. more than 50% tumour regression) was already achieved after the two induction courses; the disappearance of all lesions was observed respectively after two and four maintenance cycles. Eight patients were considered as stable disease respectively for 4+, 6, 6+, 7, 7+, 9, 11+ and 12+ months and 11 patients were progressive. Two patients classified as stable had indeed tumour regression. In one patient five pulmonary metastatic nodules disappeared whereas one residual lesion slightly increased in size (increase <25%). This patient, because of the occurrence of severe hypotension during IL2 therapy, refused to carry on treatment. He then received alpha Interferon ($18 \times 10^6$ U thrice a week) and the residual mass was reduced by approximately 75%. The status of this patient has remained stable for 11 months. In another patient two metastatic abdominal lymph nodes disappeared whereas bone lesions were stable on radiolabelled bone scan. The status of the patient has been unchanged for 12 months.

**Table V** Characteristics of the responding patients

| *Patients* | *Sex* | *Age* | *Nephrectomy* | *Tumour sites* | *Response duration (months)* |
|---|---|---|---|---|---|
| 1 | M | 69 | + | Lung/Kidney | PR (4) |
| 2 | M | 75 | + | Adrenal gland/ Abdominal mass | CR (18+) |
| 3 | M | 65 | + | Lung | CR (15+) |

PR: Partial response, CR: Complete response.

### Discussion

This is, to our knowledge, the first report of IL2 therapy in a group of patients of more than 65 years with renal cell carcinoma. Since the potential impairments or failure of different organs are of great concern in this particular population, the schedule described by West *et al.*, 1987, was used in a view to reduce toxicity. The amount of drug administered was over 70% of the planned dose in almost all the patients during the first course. Types of toxicities observed were comparable to what had been observed in younger patients and mostly manageable using symptomatic treatments and transient interruptions of rIL2 infusion. However, cardiac toxicity was of great concern; cardiac rhythm disturbances occurred in 44% of the patients and cardiac toxicity was clearly involved in the toxic death. Although cardiac toxicity of IL2 was already known and anlaysed, such a severity and incidence were not commonly reported in previous studies, specially when continuous infusion was used (Rosenberg *et al.*, 1987; West *et al.*, 1987; Fischer *et al.*, 1988; Nora *et al.*, 1989; Négrier *et al.*, 1990; Siegel *et al.*, 1991). Obviously, despite normal cardiologic tests prior to treatment, the heart of patients over 65 is particularly sensitive to IL2 toxicity. As in previous reports (Rosenberg *et al.*, 1987; West *et al.*, 1987; Fischer *et al.*, 1988; Nora *et al.*, 1989; Négrier *et al.*, 1990) fever, hypotension and renal failure were the most common other side effects encountered. They also appeared transient and limited to the treatment period.

Three responses were seen and two complete remissions were achieved i.e. two durable persistent complete responses and one partial response. In addition, among the eight patients categorised as stable, two patients also had significant persistent tumour regressions.

These results are not different from those reported in cohorts of younger patients (Rosenberg *et al.*, 1987; Fischer *et al.*, 1988; Nora *et al.*, 1989; Négrier *et al.*, 1990).

IL2 therapy which induces, according to different authors, a response rate of 20 to 30% is, in this respect, beneficial only to a minority of patients (Rosenberg *et al.*, 1987; Fischer *et al.*, 1988; Nora *et al.*, 1989; Négrier *et al.*, 1990). Nevertheless, the 5 to 10% complete response rate should not be disregarded in a disease in which nothing but rIL2 and Interferon alpha has really proved efficient (Quesada *et al.*, 1985; Bergerat *et al.*, 1988). To an individual and ethical point of view, we think we have to give a maximum of patients the chance to reach complete remission through immunotherapy. Except for some preliminary data, no predictive factor of response is known yet; and our study indicates that age does not influence the potential of tumour regression induced by *in vivo* IL2 stimulation (Blay *et al.*, 1990).

However, the schedule of treatment and the drugs to use, i.e. rIL2 *vs* IFN *vs* both of them, are still debatable. Prior studies reported interesting results with ambulatory therapy with Interferon, but the duration of these treatment schedules, i.e. 6 to 12 months, is not adapted to a disease with a crude median survival of 8 to 12 months (Ritchie *et al.*, 1987; Deforges *et al.*, 1988; Elson *et al.*, 1988; Philip *et al.*, 1989). More recently, a 'home therapy regimen' was proposed using a subcutaneous combination of rIL2 and Interferon alpha (Atzpodien *et al.*, 1990). This regimen did not raise any life threatening toxicity and the response rate obtained does not signficantly differ from those obtained with continuous infusion of IL2. To our point of view, a first line immunotherapy should be proposed to a maximum of patients in order to select those who are sensitive to this kind of therapy, and long lasting schedules should be restricted to responding patients. As such, the combined subcutaneous schedules of IL2 and IFN could represent an adapted deal to this situation. Prolonged therapy in responding patients must be evaluated in randomised trials.

In conclusion, the present study demonstrates that rIL2 continuous infusion is feasible, but has a particular cardiac toxicity in patients over 65 years old. The efficacy of rIL2 in this group is not different from what it is in the general population of patients with metastatic renal cell cancer.

### References

ATZPODIEN, J., KORFER, A., FRANKS, C.R., POLIWODA, M. & KIRCHNER, H. (1990). Home therapy with recombinant interleukin-2 and interferon α 2β in advanced malignancies. *Lancet*, **335**, 1509-1512.

BERGERAT, J.P., HERBRECHT, R., DUFOUR, P., JACQMIN, D., BOLLACK, C., PREVOT, G., BAILLY, G., DE GARIS, S., JURASCHEK, F. & OBERLING, F. (1988). Combination of recombinant Interferon Alpha and Vinblastine in advanced renal cell cancer. *Cancer*, **62**, 2320-2324.

BLAY, J.Y., FAVROT, M., NÉGRIER, S., COMBARET, V., CHOUAIB, S., MERCATELLO, A., KAEMMERLEN, P., FRANKS, C.R. & PHILIP, T. (1990). Correlation between clinical response to Interleukin-2 therapy and sustained production of tumour necrosis factor. *Cancer Res.*, **50**, 2371-2374.

DEFORGES, A., REY, A., KLINIK, A., GHOSN, M., KRAMAR, A. & DROZ, J.P. (198). Prognostic factors of adult metastatic renal carcinoma: a multivariate analysis. *Sem. Surg. Oncol.*, **4**, 149-154.

DROZ, J.P., THEODORE, C., GHOSN, M., LUPERA, H., PIOT, G., DEFORGES, A., KLINIK, M., ROUESSE, J. & AMIEL, J.L. (1988). Twelve-year experience with chemotherapy in adult metastatic renal cell carcinoma at the Institut Gustave Roussy. *Sem. Surg. Oncol.*, **4**, 97-99.

ELSON, P.J., WITTER, S. & TRUMP, D.L. (1988). Prognostic factors for survival in patients with recurrent or metastatic renal cell carcinoma. *Cancer Res.*, **48**, 7310-7313.

FINNEY, R. (1973). An evaluation of postoperative radiotherapy in hypernephroma treatment - a clinical trial. *Cancer*, **32**, 1332-1340.

FISCHER, R.I., COLTMAN, C.A., DOROSHOW, J.H., RAYNER, A.A., HAWKINS, M.J., MIER, J.N., WIERNICK, P., MAC MANNIS, J.D., WEISS, G.R., MARGOLIN, K.A., GEMLOB, B.T., HOTH, D.F., PARKINSON, D.R. & PAIETTA, E. (1988). Metastatic renal cell cancer treated with IL2 and lymphokine activated killer cells. A phase II trial. *Ann. Int. Med.*, **108**, 518-523.

HOLLAND, J.M. (1973). Cancer of the Kidney-Natural history and staging Cancer. **32**, 1030-1042.

MILLER, A.B., HOOGSTRATEN, B., STAQUET, M. & WINKLER, A. (1987). Reporting results of cancer treatment. *Cancer*, **47**, 207-214.

NÉGRIER, S., PHILIP, T., STOTER, G., FOSSA, S.D., JANSSEN, S., IACONE, A., CLETON, F.S., EREMIN, O., ISRAEL, J., JASMIN, C., RUGARLI, C., MASSE, H.V.D., THATCHER, N., SYMANN, M., BARTSCH, M.M., BERGMANN, L., BIJMAN, J.T., PALMER, P.A. & FRANKS, C.R. (1990). Interleukin-2 (IL2) with or without LAK Cells in metastatic renal cell carcinoma: a report of a European multicentre study. *Eur. J. Cancer Clin. Oncol.*, **25**, 21-28.

NORA, R., ABRAMS, J.S., TAIT, N.S., HIPONIA, D.J. & SILVERMAN, H.K. (1989). Myocardial toxic effects during rcombinant interleukin-2 therapy. *J. Natl Cancer Inst.*, **81**, 59-63.

PHILIP, T., MERCATELLO, A. NÉGRIER, S., PHILIP, I., REBATTU, P., KAEMMERLEN, P., GASPARD, M., TOGNET, E., COMBARET, V., BIJMAN, J.T., FRANKS, C.R., CHAUVIN, F., MOSKOVTCHENKO, J.F., FAVROT, M. & CLAVEL, M. (1989). Interleukin-2 with or without LAK cells in metastatic renal cell carcinoma: the Lyon first year experience on 20 patients. *Cancer Treat. Rev.*, **16**, 91-104.

QUESADA, J.P., RIOS, A., SWANSON, D., TROWN, P. & GUTTERMAN, J.U. (1985). Antitumor activity of recombinant derived interferon α in metastatic renal cell carcinoma. *J. Clin. Oncol.*, **3**, 1522-1528.

RITCHIE, A.W.S. & DE KERNION, J. (1987). The natural history and clinical features of renal carcinoma. *Sem. Nephrol.*, **7**, 131-139.

ROSENBERG, S.A., LOTZE, M.T., MUUL, L.M., CHANG, A.E., AVIS, F.P., LEITMAN, S., LINEHAN, W.M., ROBERTSON, C.N., LEE, R.E., RUBIN, J.T., SEIPP, L.A., SIMPSON, C.G. & WHITE, D.E. (1987). A progress report on the treatment of 157 patients with advanced cancer using lymphokin-activated killer cells and interleukin-2 or high-dose interleukin-2 alone. *New Engl. J. Med.*, **316**, 889-897.

SIEGEL, J.P. & PURI, R.K. (1991). Interleukin-2 toxicity. *J. Clin. Oncol.*, **9**, 694-704.

WEST, W.H., TAUER, K.W., YANELLI, J.R., MARSHALL, G.D., ORR, D.W., THURMAN, G.B. & OLDHAM, R.K. (1987). Constant-infuson recombinant Interleukin-2 in adoptive immunotherapy of advanced cancer. *New Engl. J. Med.*, **316**, 898-905.


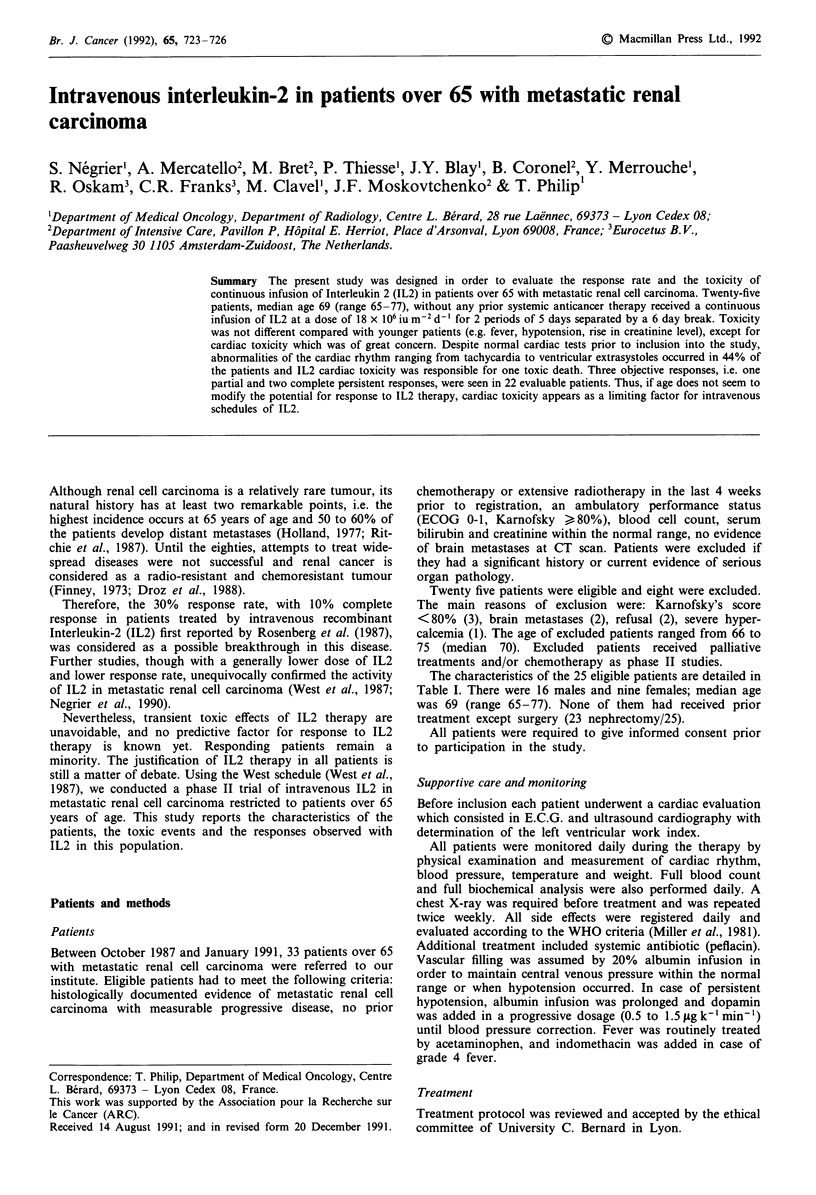

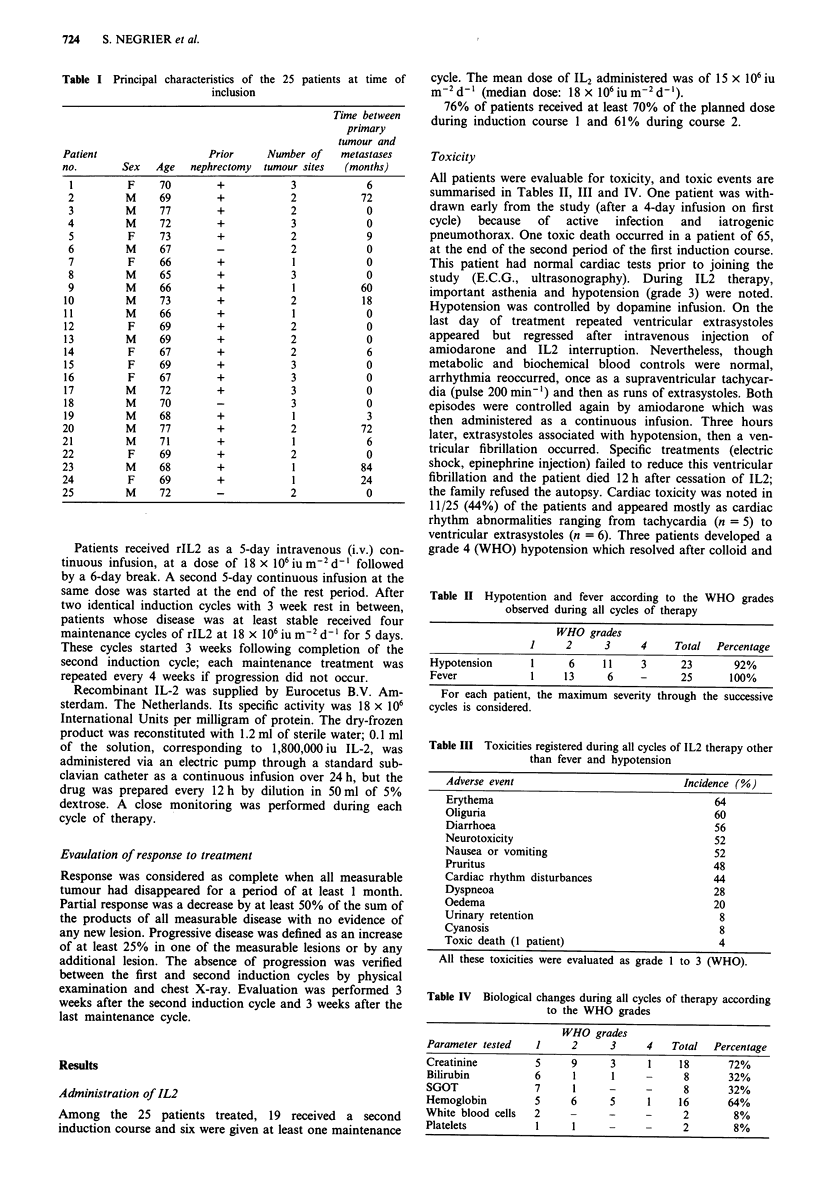

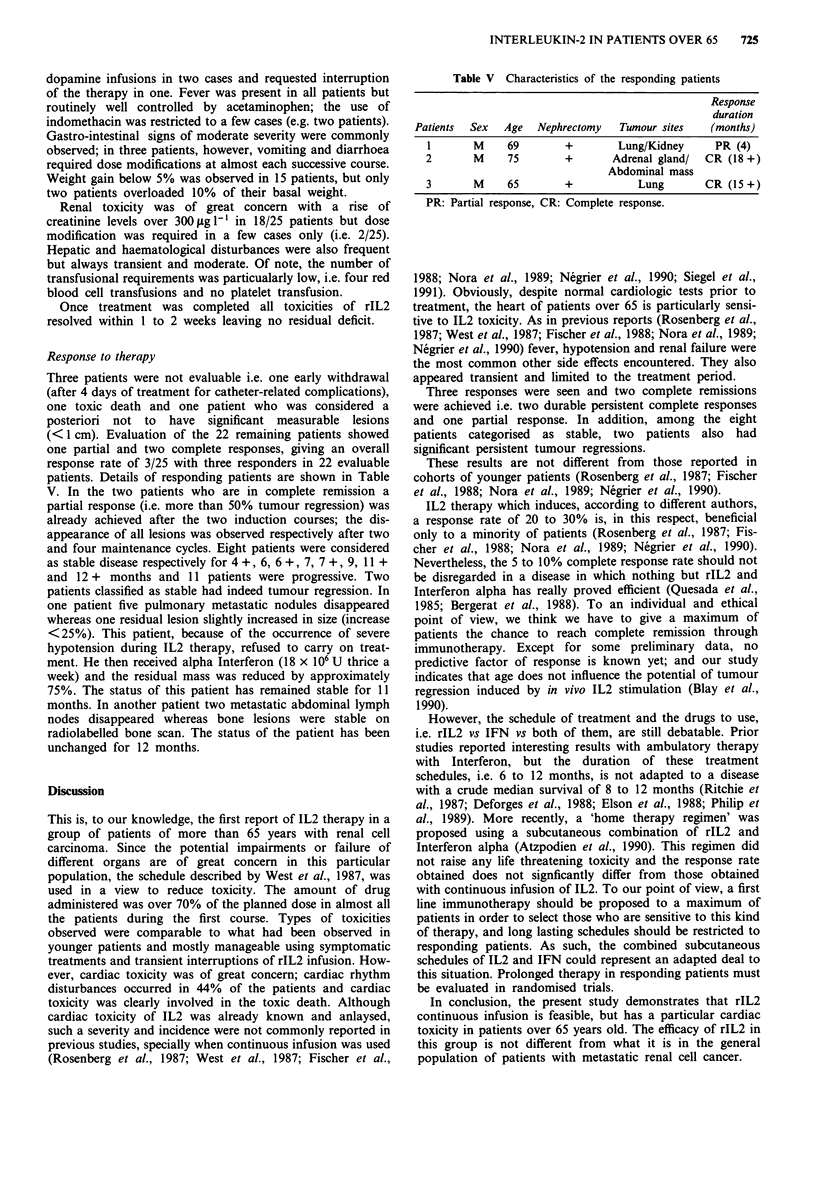

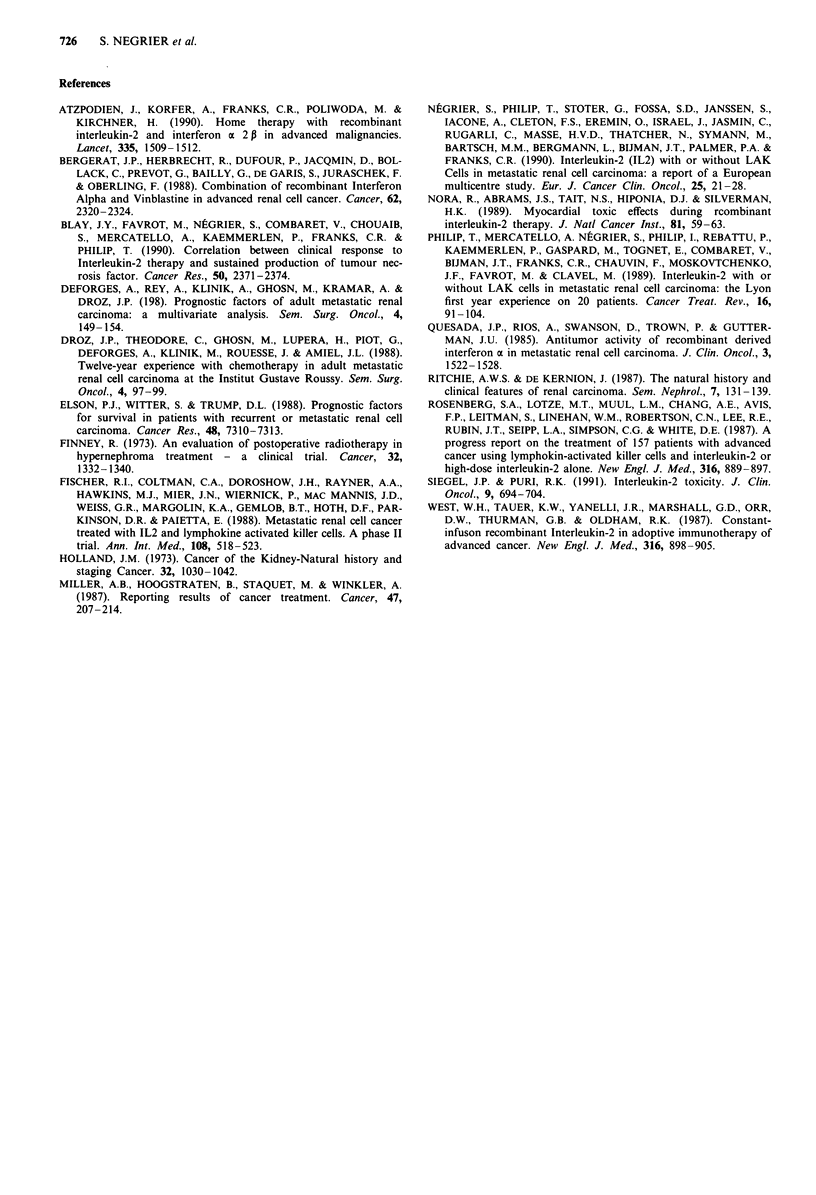


## References

[OCR_00588] Atzpodien J., Körfer A., Franks C. R., Poliwoda H., Kirchner H. (1990). Home therapy with recombinant interleukin-2 and interferon-alpha 2b in advanced human malignancies.. Lancet.

[OCR_00596] Bergerat J. P., Herbrecht R., Dufour P., Jacqmin D., Bollack C., Prevot G., Bailly G., de Garis S., Juraschek F., Oberling F. (1988). Combination of recombinant interferon alpha-2a and vinblastine in advanced renal cell cancer.. Cancer.

[OCR_00601] Blay J. Y., Favrot M. C., Negrier S., Combaret V., Chouaib S., Mercatello A., Kaemmerlen P., Franks C. R., Philip T. (1990). Correlation between clinical response to interleukin 2 therapy and sustained production of tumor necrosis factor.. Cancer Res.

[OCR_00614] Droz J. P., Theodore C., Ghosn M., Lupera H., Piot G., De Forges A., Klink M., Rouesse J., Amiel J. L. (1988). Twelve-year experience with chemotherapy in adult metastatic renal cell carcinoma at the Institut Gustave-Roussy.. Semin Surg Oncol.

[OCR_00621] Elson P. J., Witte R. S., Trump D. L. (1988). Prognostic factors for survival in patients with recurrent or metastatic renal cell carcinoma.. Cancer Res.

[OCR_00626] Finney R. (1973). An evaluation of postoperative radiotherapy in hypernephroma treatment--a clinical trial.. Cancer.

[OCR_00635] Fisher R. I., Coltman C. A., Doroshow J. H., Rayner A. A., Hawkins M. J., Mier J. W., Wiernik P., McMannis J. D., Weiss G. R., Margolin K. A. (1988). Metastatic renal cancer treated with interleukin-2 and lymphokine-activated killer cells. A phase II clinical trial.. Ann Intern Med.

[OCR_00639] Holland J. M. (1973). Proceedings: Cancer of the kidney--natural history and staging.. Cancer.

[OCR_00643] Miller A. B., Hoogstraten B., Staquet M., Winkler A. (1981). Reporting results of cancer treatment.. Cancer.

[OCR_00657] Nora R., Abrams J. S., Tait N. S., Hiponia D. J., Silverman H. J. (1989). Myocardial toxic effects during recombinant interleukin-2 therapy.. J Natl Cancer Inst.

[OCR_00664] Philip T., Mercatello A., Negrier S., Philip I., Rebattu P., Kaemmerlin P., Gaspard M., Tognier E., Combaret V., Bijmann J. T. (1989). Interleukin-2 with and without LAK cells in metastatic renal cell carcinoma: the Lyon first-year experience in 20 patients.. Cancer Treat Rev.

[OCR_00673] Quesada J. R., Rios A., Swanson D., Trown P., Gutterman J. U. (1985). Antitumor activity of recombinant-derived interferon alpha in metastatic renal cell carcinoma.. J Clin Oncol.

[OCR_00677] Ritchie A. W., deKernion J. B. (1987). The natural history and clinical features of renal carcinoma.. Semin Nephrol.

[OCR_00680] Rosenberg S. A., Lotze M. T., Muul L. M., Chang A. E., Avis F. P., Leitman S., Linehan W. M., Robertson C. N., Lee R. E., Rubin J. T. (1987). A progress report on the treatment of 157 patients with advanced cancer using lymphokine-activated killer cells and interleukin-2 or high-dose interleukin-2 alone.. N Engl J Med.

[OCR_00687] Siegel J. P., Puri R. K. (1991). Interleukin-2 toxicity.. J Clin Oncol.

[OCR_00691] West W. H., Tauer K. W., Yannelli J. R., Marshall G. D., Orr D. W., Thurman G. B., Oldham R. K. (1987). Constant-infusion recombinant interleukin-2 in adoptive immunotherapy of advanced cancer.. N Engl J Med.

